# What to Do with Backward Children

**Published:** 1913-01

**Authors:** F. S. White

**Affiliations:** Superintendent Southwestern Insane Asylum, San Antonio, Texas


### What to Do With Backward Children.

BY F. S. WHITE, M. D., SUPERINTENDENT SOUTHWESTERN INSANE ASYLUM, SAN ANTONIO, TEXAS.

I shall deal in plain words, leaving out as far as possible all technical terms. When one has been engaged in the practice of medicine for over a quarter of a century, he learns to say what he has to say in the fewest and plainest words.

I am to talk to you today on "What to Do With Backward Children." This is a great problem—a problem that has engaged in the past and is engaging today, the best thought and efforts of some of the most profound and philanthropic thinkers of the world.

Upon the solution of this problem, to a very considerable extent, rests the future of the human race and determines the progress of civilization—and mental and moral development.

The world is ruled by intelligence, and intelligence is the result of normal, well-developed brain matter. Without normal intellectual and physical development, no race of people can survive, much less make development.

Backward children may be divided into three main classes:

First—Idiots, who occupy the lowest grade, and as a rule are not capable of much improvement.

Second—Imbeciles, who are a little above idiots in point of intelligence and capability.

Third—The feeble-minded, who are usually capable of acquiring considerable learning of a rudimentary kind, and who often make useful members of a community.

There are several subdivisions of these main classes, but I do not think it necessary or advisable to take your time to enumerate them.

Now, the question that looms large before us is, What can be done with mental weaklings?

When physicians take up the consideration of a disease, the first things to be determined are the causes, then the prevention; and last the treatment; and in this order I shall discuss this subject. I shall consider all these classes of mental defectives together as combining to form the class which we designate as "backward children."

Among the great number of causes which produce this condition, a few stand out very prominently. Injuries before birth, at birth and those during the first few years of life are responsible for a considerable number. Under this head I include not only those injuries produced by external violence, such as falls, etc., but those injuries to the brain due to acute diseases, such as meningitis, scarlet fever, diphtheria, etc.

The *modus operandi* of these agencies cannot be definitely explained, but in some way they serve to arrest the development of the brain. The great cause, and the one most prolific of results, is that of heredity—that mysterious something which pervades and controls both the animal and vegetable kingdoms, and by virtue of which "like begets like."

La Page says that "a family taint of mental deficiency, insanity or epilepsy, constituting what is known as the neuropathic inheritance, is the underlying cause of primary feeble-mindedness in 90 per cent of all cases."

This imperative and potential force is that which accounts for the perpetuation of both animal and human species. By taking advantage of it, the stockman improves his breed until apparently perfection is reached.

Without this miraculous and divine force there could have been no breed of race horses; there could have been no Berkshire hogs; there could have been no Jersey or Holstein cattle, no Plymouth Rock chickens, Maltese cats or poodle dogs; there could have been no delicious tomatoes; there could have been no pumpkin yam potatoes, no stately and magnificent geraniums, or lovely and delightful violets, no Marshal Neil or La France roses, no trailing arbutus or enchanting orchids to grace my lady's table.

I could pursue these illustrations indefinitely and multiply examples to show you how this great force runs through the universe, and how shrewd and far-seeing men have taken advantage of it to multiply their fortunes. I want to say to you, without doubt or hesitation, that this great power must be invoked and utilized in order that the human race may reach that supreme eminence for which it was intended.

By omniscient divinity this great power has been given to man, but through ignorance or cupidity he has failed to utilize it in the upbuilding of the race to the extent to which it is capable.

It is true that, in spite of the violation of almost every law of nature, man has continued to improve and advance, until he is today nearer perfection than ever before, but yet is a long way from the goal.

When a man selects the sire for the future offspring of his herds, he is very careful to select only those of the purest strain and whose pedigree is without a blemish; but when he aids in the selection of the father for his grandchildren any cane-sucking scion of luetic ancestry will do.

I consider that in this day of enlightenment it is a reproach and disgrace upon any man or woman to be the father or mother of idiots or defective children when the cause is heredity.

Why this false modesty? Why cannot men and women devote as much reason and thought to the breeding of people as they do to the breeding of cattle, sheep and dogs?

It is certainly a million times more honor and credit to produce one magnificent, fine, fat, crowing baby than all of the Maltese cats in Christendom. The grandest possession that can honor and grace your magnificent homes is rosy, romping, healthy boys and girls—they beat bird dogs, canary birds and poll parrots all hollow. There is something to a rollicking, riproaring boy; there is nothing to a high-bred poodle.

Now, ladies, permit me to urge upon you that when you think about parents for your grandchildren, you bear in mind this great, cold-blooded, unmerciful and inviolable law of heredity. If you do not, then in future years the effects of its violation may rise up and plague you. And allow me to impress this great truth upon you, that the grandest heritage that can be bequeathed to your offspring is a sound mind in a sound body.

In order that growth and development may be normal, the growing child must have an abundance of nourishing food; he must have fresh air and sunshine; he must get close to Mother Earth and imbibe the life-giving principle that emanates from her bosom.

Plain, clean dirt will not hurt your darling boy or angel girl—unless you deprive them of it. It is just as natural for a child to want to play in the dirt as it is for a duck to swim. Give a little tot all dressed in spotless white an opportunity, and he drops on the ground to sift sand through his fingers.

If you would have your child healthy and robust, let him out on the ground where God's fresh air and sunshine can paint his cheek's with brushes made of sunbeams and dipped in the colors which make nature's flowers reflect the beauties of the divine.

Let them out where they may receive the benefit of the life-giving essence which pervades all nature and adds to the red blood coursing through their veins—that which makes bone, brawn and brain. After a day thus spent, and when sleep, nature's sweet restorer, is knitting up the raveled sleeve, they will have visions of angels and hear celestial music, instead of being racked with colic and haunted by hobgoblins. These are some of the things that prevent backwardness in children.

I have endeavored to tell you how to prevent the production of backward children, and will now consider the care of those who, like the poor, are always with us.

Idiots and imbeciles, except the higher grades of the latter class, must be cared for in institutions especially prepared for them. They cannot fight the battle of life unaided and alone. To a very limited extent their work can be utilized to aid in their support. They can be taught to do many kinds of plain, unskilled labor, such as farming and gardening, but must be continually under the control and direction of others. They have no initiative, and are very defective in reasoning power.

This class of feeble-minded children are such as enter school and soon fall behind, do not make progress as other children, and rarely pass the sixth or seventh grades.

These are the children that need expert training, which, to be effective in shaping their characters, must be begun early. It requires great patience and adaptability to this kind of work in teachers in order that the best results may be obtained.

Many of this class of backward children can, by the right training, be started on the road to further development, and eventually make useful citizens. Some of them, after arriving at puberty, take on a new growth, both bodily and mentally, and sometimes outgrow the earlier defects.

In some of the larger schools of the country, special expert teachers are employed for the backward children, and so adroitly are they managed that they are not humiliated by the fact that they are not the equal of the other children, and, in fact, do not realize that they are receiving any special attention because they are backward.

Self-respect must be maintained, and healthy efforts stimulated. Their capacity must be closely investigated and such lines of work required as they are capable of accomplishing.

A great many children are irretrievably injured in schools by being required to do more work than they are capable of. There is a limit to mental work, just as there is a limit to physical work, and it is no more reasonable to expect every child of a given age to do the same amount of mental work than it is to require them to do the same amount of physical work.

Ambitious parents often overtax the child, causing an arrest of mental growth, with a resultant feeble-minded child, whose mind, under other conditions, would have gone on developing in a normal manner.

Teachers should study closely the potential capacity of each child confided to their care. This arrest of growth by overstudy in school often results in a complete mental breakdown in later years, and the unfortunate victim finally reaches a hospital for insane. I have had several girls under my care whose condition was easily traceable to overwork at school—to attempt to do work beyond their capacity. I often think that it is a mistake made by our public schools to attempt to bring all the children of about the same age up to the same standard.

Would it not be more rational to classify children according to mental capacity rather than according to age? This, to be sure, is done to some extent, but often a very dull child is severely injured in the attempt to keep up with a very bright child.

Now, in conclusion, I desire to summarize what I have said, in order that the main points may be emphasized.

Mentally defective children are divided into three main classes—idiots, imbeciles and feeble-minded.

Idiots and imbeciles are incapable of acquiring a very high degree of learning, and, as a rule, when once placed in an institution for their care they remain there the balance of their lives, as they are incapable of fighting the battle of life alone.

As a great aid in reducing the number of feeble-minded children, advantage should be taken of fresh air and sunshine and outdoor life.

Many cases of feeble-mindedness are caused by an attempt on the part of the children to do work for which they are incapacitated. This work is usually imposed upon them by ambitious parents.

The higher class of the feeble-minded or backward children should have special trained teachers who know how to train their weakened and crippled minds and stimulate a new growth and development.

The cause which accounts for 90 per cent of all feeble-minded children is that great force in nature which we call heredity, and it is the power which, if recognized and utilized in the perpetuation of people, would in a few generations almost eliminate the feeble-minded.

A little while ago I looked upon a magnificent picture which reflected the handiwork of Deity. It adorned the front page of a publication devoted exclusively to agriculture and stock raising. This picture represented, in all his grandeur, a magnificent animal which had been produced by a strict observance for generations of the law of heredity, and demonstrated what can be done with one of the meanest species of the brute creation. The progeny of this splendid specimen has added untold wealth to this country. Astride this animal was a curly-headed, laughing boy, and I found myself wondering whether or not as much thought and consideration had been given to the production of the one as to the other.

---

WE CALL ATTENTION to an announcement in this issue of "The Correspondence School of Gospel and Scientific Eugenics." Write for the catalogue; it will cost you nothing. Mrs. Mary Teats, the principal and founder of this correspondence school, should be numbered among the world's noblest benefactors, for most surely all great reforms must begin with the fathers and mothers. I have looked carefully over the school curriculum, which embraces five grades, and I am delighted. I wish every mother in Texas could take this course of instruction. The terms are very reasonable.

---

OUTLINE FOR CLUB STUDY for January:

Bible reading—Isaiah 40:28-31.

Roll call; responses on "Physical Purity."

Reading of minutes.

Paper—"Qualifications for Parenthood." (Published herewith.)

Benediction—I. Chron., 39:11-13.

---

CLUB: We will send the RED BACK one year, Uncle Remus Home Magazine six months, Southern Ruralist one year, Gentlewoman one year, Good Stories one year—all for $1.50—cash with order. Samples of the last four publications will be sent free by the Uncle Remus Home Magazine, Atlanta, Ga.

### QUESTION BOX.

At what age is cancer of the uterus most apt to begin, and what are the first signs?

Cancer is most apt to develop at the time of the menopause, or change of life. It may occur as early as thirty years, but this is very unusual. The danger signals are (1) a reappearance of the flow after it has ceased for several months; (2) a bloody discharge between periods; (3) a prolonged and profuse menstrual flow.

M. H.

Is it necessary to put flannels on babies?

Yes; every new-born baby should have a band and shirt of flannels or flannel and silk mixed. In the winter a flannel skirt should be added. Flannel should be kept over the abdomen until after the second summer if the child is at all delicate or has trouble while teething. The flannel should be of light weight. M. H.

